# Functional Characterization of *Saccharomyces cerevisiae* P5C Reductase, the Enzyme at the Converging Point of Proline and Arginine Metabolism

**DOI:** 10.3390/microorganisms10102077

**Published:** 2022-10-20

**Authors:** Giuseppe Forlani, Giuseppe Sabbioni, Milosz Ruszkowski

**Affiliations:** 1 Department of Life Science and Biotechnology, University of Ferrara, 44121 Ferrara, Italy; 2 Department of Structural Biology of Eukaryotes, Institute of Bioorganic Chemistry, Polish Academy of Sciences, 61-704 Poznan, Poland

**Keywords:** P5C reductase, proline, arginine, allosteric regulation, NAD(P)H as electron donor

## Abstract

The enzyme that, in *Saccharomyces cerevisiae,* catalyzes the last step in both proline synthesis and arginine catabolism, δ^1^-pyrroline-5-carboxylate (P5C) reductase, was purified to near homogeneity and characterized thoroughly. Retention patterns upon gel permeation chromatography were consistent with a homodecameric composition of the holomer. High lability of the purified preparations and stabilization by reducing compounds suggested susceptibility to reactive-oxygen-species-mediated damage. Both NADH and NADPH were used as the electron donor, the latter resulting in a 3-fold higher V_max_. However, a higher affinity toward NADH was evident, and the NADPH-dependent activity was inhibited by NAD^+^, proline, arginine, and a variety of anions. With proline and arginine, the inhibition was of the competitive type with respect to the specific substrate, and of the uncompetitive- or mixed-type with respect to NADPH, respectively. The results suggest that, contrary to the enzyme from higher plants, yeast P5C reductase may preferentially use NADH in vivo. An in silico analysis was also performed to investigate the structural basis of such enzyme features. Superposition of the protein model with the experimental structure of P5C reductase from *Medicago truncatula* allowed us to hypothesize on the possible allosteric sites for arginine and anion binding, and the cysteine pairs that may be involved in disulfide formation.

## 1. Introduction

Due to its peculiar properties, in recent decades, the metabolism of the cyclic amino acid proline has attracted increasing interest. In addition to its role as a proteinogenic compound, in most microorganisms and plants, proline acts as a stress protectant, being accumulated in the cell to high concentrations in response to a plethora of stress conditions, ranging from drought to excess salt, and from cold and freezing to the presence of heavy metals [1]. The exact mechanisms by which proline benefits the cell are still a matter of debate, and it has been hypothesized that it may act as a compatible osmolyte favoring osmotic compensation, as a protein-folding chaperone in the presence of high salt levels, and as a membrane stabilizer or a scavenger of reactive oxygen species (ROS) [2]. Moreover, the interconversion of glutamate and proline is believed to contribute to controlling the redox status and the NADP(H) to NAD(H) ratio in the cell, and to allow transferring reducing equivalents to the mitochondrial respiratory chain [3]. As a consequence, modulation of proline metabolism is required both in plants for the activation of the hypersensitive response to pathogen attack [4], and in mammals to alternatively lead to ROS production and triggering of the apoptotic mechanism, or to increased ATP synthesis for protective autophagy [5]. Consistently, growing evidence shows that the enzymes involved in proline metabolism are subject to a complex array of regulative mechanisms at both the transcriptional [6,7] and post-translational [8,9] levels.

In this context, the budding yeast *Saccharomyces cerevisiae* represents an exception, as it does not accumulate proline under the above-mentioned stress conditions [10]. Proline in yeast is synthesized in the cytosol from glutamate through three steps catalyzed by γ-glutamyl-kinase (Pro1, EC 2.7.2.11), γ-glutamylphosphate reductase (Pro2, EC 1.2.1.41), and δ^1^-pyrroline-5-carboxylate (P5C) reductase (Pro3; EC 1.5.1.2) [11]. The last reaction is shared with the pathway for arginine degradation [12], in which P5C is produced by ornithine-δ-aminotransferase (OAT, EC 2.6.1.13). Because of this, strains that are defective for pro1 and pro2 are able to satisfy their requirement for proline through arginine catabolism. However, when preferred nitrogen sources (such as ammonia, glutamine, or asparagine) are present, the arginine degradation pathway is blocked by the nitrogen catabolite repression system (NCRs) [13]. As a consequence, pro1 and pro2 strains can grow only in minimal media lacking ammonia, in which the NCRs is not activated. Proline homeostasis is ensured by transcriptional control of *Pro1* and *Pro2*, as well as by negative feedback inhibition of Pro1 activity. A Pro1 variant that was less sensitive to feedback inhibition was found to accumulate proline, and exhibited an increased tolerance to freezing and desiccation [14]. High levels of proline induce, in turn, the expression of the proline catabolic pathway, which is located in the mitochondrion and consists of two steps sequentially catalyzed by proline dehydrogenase (Put1, EC 1.5.5.2) and P5C dehydrogenase (Put2, EC 1.2.1.88) [15]. *Put1* and *Put2* are induced by a transcriptional activator, Put3, which is constitutively bound to *Put1* and *Put2* promoters, but is maximally activated only in the presence of proline and in the absence of better nitrogen sources [16]. Moreover, *Pro1* and *Pro2* expression is upregulated by the general amino acid control, a system by which, in yeast, many of the genes involved in amino acid biosynthesis are induced in response to starvation for any single amino acid [17]. In striking contrast, *Pro3* is constitutively expressed and is—apparently—under no form of regulation.

Yeast P5C reductase was purified and partially characterized in early studies [18,19]. The enzyme was found to use either NADH or NADPH as the electron donor with similar affinity, but resulting in twice the maximal velocity with the former. The native relative molecular mass (about 120 kDa) was consistent with an unusual tetrameric quaternary structure. To the best of our knowledge, no further studies have investigated enzyme features, and the possible occurrence of mechanisms for post-translational regulation has not been assessed. Indeed, recent data showed that the enzyme from other sources is subject to allosteric modulation by proline, NAD(P)^+^, and salts [20,21,22,23], with remarkable differences depending on which reduced pyridine dinucleotide acts as a co-factor [8,24]. Here, we report a thorough characterization of *S. cerevisiae* P5C reductase, as well as an in silico analysis to compare the structure and the properties of the enzyme from yeast with those from plants, mammals, and bacteria.

## 2. Materials and Methods

### 2.1. Substrates and Reagents

Unless otherwise specified, all reagents were purchased from Merck (Darmstadt, Germany), and were of analytical grade. Δ^1^-pyrroline-5-carboxylic acid was synthesized by the periodate oxidation of δ-*allo*-hydroxylysine (Sigma H0377) and purified by cation-exchange chromatography onto a Dowex AG50W-X4 (200–400 mesh) column, as described in [25]. DL-P5C solutions (about 50–60 mM) in 1 M HCl were stored at 4 °C in the dark, and brought to neutral pH just before the assay using proper aliquots of a 1 M Tris base solution.

### 2.2. Purification of Yeast P5C Reductase

Commercial bakers’ yeast (5 to 10 g material) was homogenized in an ice-cold mortar with 4 g g^−1^ of alumina and resuspended in 5 mL g^−1^ of extraction buffer (50 mM Tris-HCl buffer, pH 7.5, containing 1 mM dithiothreitol [DTT]). All subsequent operations were carried out at 4 °C. The homogenate was clarified for 5 min at 3000× *g*, and the supernatant was further centrifuged for 5 min at 12,000× *g*. The crude extract was fractionated with ammonium sulfate, and the 60–80% saturated fraction was collected by centrifugation, resuspended in 5–10 mL of extraction buffer, and desalted by passage through a BioGel P6DG column equilibrated with the same buffer. The desalted sample was loaded at a constant flow of 60 mL h^−1^ onto a DEAE-Sephacel column (2.5 cm ϕ; 30 mL bed volume). Proteins were eluted with a linear gradient from 0 to 300 mM NaCl in a 400 mL buffer while collecting 8 mL fractions. Active fractions were pooled and immediately supplemented with 1 mM DTT. The sample was concentrated to a volume of about 5 mL by centrifugation in a centrifugal filter device with a cut-off of 50 kDa (Amicon Ultra-15, Millipore [Burlington, MA, USA]), and loaded onto a Sephacryl S300 (Pharmacia [Cologno Monzese, Italy]) column (2.5 cm ϕ; 250 mL bed volume) equilibrated in column buffer (50 mM Tris-HCl buffer, pH 7.5, containing 1 mM DTT and 200 mM NaCl). Elution proceeded at a flow rate of 30 mL h^−1^, for the collection of 2.5 mL fractions. Active fractions were pooled, concentrated as above, and column-desalted against extraction buffer, as described.

Discontinuous SDS–polyacrylamide gel electrophoresis was performed at 8 mA with a 5% stacking and a 12% separating gel, using a Minigel system (BioRad [Segrate, Italy]). Samples were mixed with the same volume of 125 mM Tris-HCl buffer (pH 6.8) containing 4% (*w*/*v*) SDS, 20% (*v*/*v*) glycerol, and 10% (*v*/*v*) β-mercaptoethanol, and denatured 5 min at 100 °C. Proteins were visualized by soaking gels in Quick Coomassie Stain (CliniSciences [Nanterre, France]) overnight.

### 2.3. P5C Reductase Assay

Enzyme activity was measured at 30 °C as the P5C-dependent oxidation of NAD(P)H. Assays were performed in 96-microwell plates in a final volume of 0.2 mL. The standard assay mixture contained 2 mM DL-P5C and 0.5 mM of either NADH or NADPH in 25 mM Tris-HCl buffer, pH 7.2. Parallel blanks were performed in which P5C had been omitted. The decrease in absorbance was measured for 5 min at 0.5 min intervals using a Ledetect plate reader (Labexim [Lengau, Austria]) equipped with an LED plugin at 340 nm. Residual NAD(P)H content was estimated on the basis of calibration curves obtained under the same conditions. Activity was calculated by linear regression of data using Prism 6 for Windows, version 6.03 (GraphPad Software [San Diego, CA, USA]). Protein concentration was measured by the Coomassie Brilliant Blue method [26], using bovine serum albumin (BSA) as the standard. To evaluate substrate affinity, invariable substrates were fixed at the same levels as in the standard assay. For variable substrates, L-P5C ranged from 35 to 1000 μM, while the concentration of NADH and NADPH ranged from 50 to 500 μM. In all cases, assays were performed in triplicate (technical replications). Each experiment was repeated at least twice with different enzyme preparations (biological replicates). K_M_ and V_max_ values, as well as K_I_s and their confidence intervals, were estimated by nonlinear regression analysis using Prism 6. The concentrations causing 50% inhibition (IC_50_) and their confidence intervals were computed by using the function log(inhibitor) vs. normalized response—variable slope.

### 2.4. In Silico Analyses

The three-dimensional model of *Sc*P5CR was built using the ColabFold implementation of AlphaFold [27]. Residue conservation on the enzyme surface was calculated using the Consurf server [28]. Identity cut-offs were set to 35–95%, which gave 1991 unique sequences from the Uniref90 subset [29] that were compared, and the conservation scores were calculated on a sample of 150 sequences that represent the list of homologs to the query. Structural analyses and molecular figures were conducted in UCSF Chimera 1.16 [30]. The charge distribution on the protein surface was calculated in the *PDB2PQR* and *APBS* servers [31,32].

## 3. Results

### 3.1. Purification of Yeast P5C Reductase

**Figure 1:**
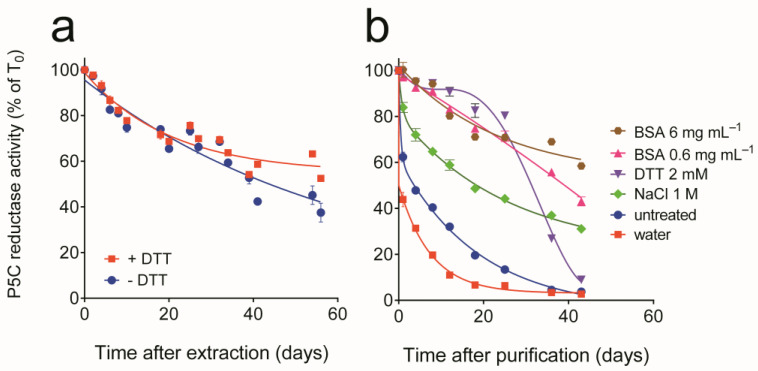
Stability of yeast P5C reductase in crude extracts and in purified preparations. (**a**) Crude extracts were prepared in the presence or in the absence of 1 mM DTT, and stored on ice; the specific activity of the enzyme was measured at increasing time, and expressed as percent value of activity at time 0 (102.7 ± 0.7 nkat mg^−1^ protein, evaluated using NADPH as the electron donor). Two different experiments were performed, each with 3 replicates. Data were combined and plotted as mean values ± SE. (**b**) Active fractions from a DEAE-Sephacel column were pooled; aliquots were mixed with the same volume of different solutions, as indicated, and stored on ice. The specific activity of the enzyme was measured at increasing time and expressed as percent value of activity at time 0 (48.95 ± 0.37 μkat mg^−1^ protein). Data are mean ± SE over 3 independent replicates.

When assayed using NADH as the electron donor, P5C reductase activity was detectable in extracts from commercial bakers’ yeast at a relatively constant level of 41.6 ± 1.4 nkat mg^−1^ protein (n = 18). The activity in crude extracts was quite stable at 4 °C, with a half-life of about 50 days. The addition of a reducing compound was neither required nor enhanced the resulting activity level, yet the presence of 1 mM DTT was found to slightly increase enzyme stability with time (Figure 1a). This notwithstanding, when, during preliminary experiments, crude extracts were fractionated by anion-exchange chromatography, activity was rapidly lost in partially purified preparations. The assessment of the effect of treatments with potentially protective compounds confirmed enzyme lability in diluted preparations. The activity was stabilized by adding substances able to scavenge reactive oxygen species, such as BSA and DTT (Figure 1b). Therefore, DTT was included in all purification steps, and allowed the attainment of homogeneous preparations (Supplementary Figure S1) through a combination of ammonium sulfate fractionation, anion-exchange, and gel permeation chromatography (Table 1).

**Figure 2:**
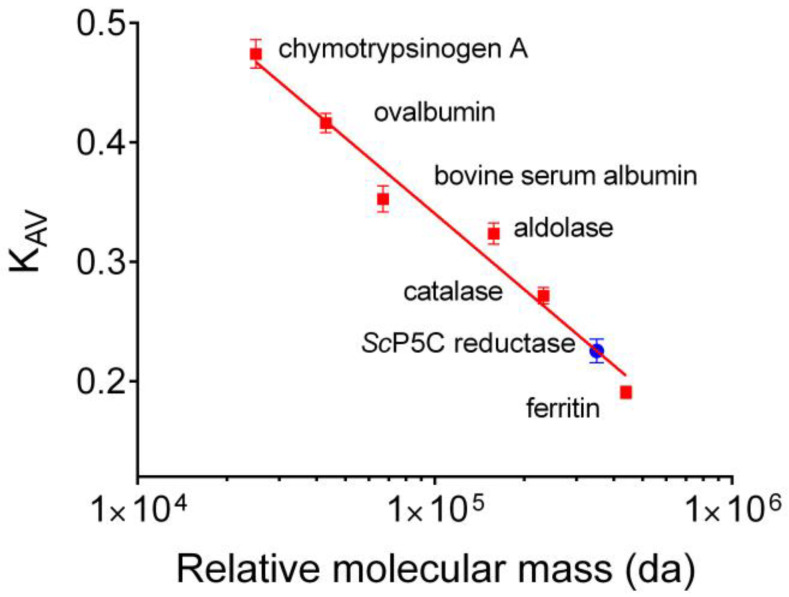
Relative molecular mass of yeast P5C reductase under native conditions. The retention pattern of the enzyme upon gel permeation chromatography on a Sephacryl S300 column was compared with that of molecular weight markers (Gel Filtration Calibration Kit, Pharmacia 17-0442-01 and 17-0441-01). Results are mean ± SD over 3 independent runs. Linear regression of K_AV_ values plotted against the logarithm of the protein molecular mass allowed an estimate of 320 ± 15 kDa for yeast P5C reductase.

The last step also allowed an estimate of the relative molecular mass of the enzyme under native conditions. The comparison with the elution pattern of molecular markers (Figure 2) was consistent with a mass of 320 ± 15 kDa. Based on a gene-deduced molecular mass of 30,132 Da for a single subunit, this suggested a homodecameric composition of the holomer. Specific activity in final samples reflected a 2500-fold purification, with a yield of about 16% of the initial amount of the enzyme.

### 3.2. Properties of Yeast P5C Reductase

The purified enzyme was characterized thoroughly. The results are summarized in Table 2. As expected, yeast P5C reductase was able to use either pyridine dinucleotide as the electron donor. The apparent affinity for NADH was higher than that for NADPH, yet the maximal activity attainable with the former was 3-fold lower than with the latter. With respect to the specific substrate also, the apparent affinity for P5C was 5-fold higher when NADH was the co-factor, but under non-limiting conditions, the maximal activity was 2-fold higher with NADPH (Supplementary Figure S2). As a consequence, activities under standard assay conditions were comparable.

**Figure 3:**
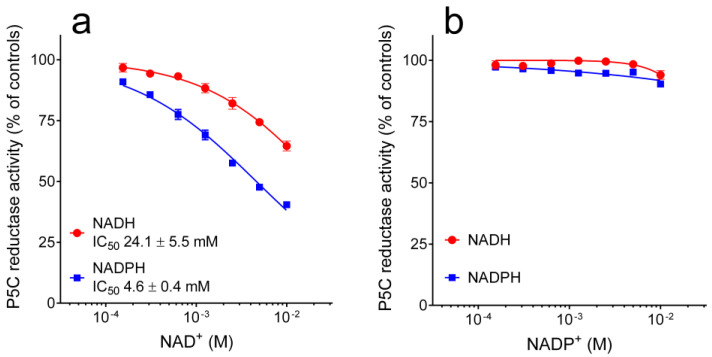
Effect of oxidized pyridine dinucleotides on the activity of yeast P5C reductase. The purified enzyme was assayed in the absence or in the presence of increasing concentrations of NAD^+^ (**a**) or NADP^+^ (**b**), using either NADH or NADPH as the electron donor. Results were expressed as percent of the activity obtained with untreated controls. Data are means ± SE over three replicates.

**Figure 4:**
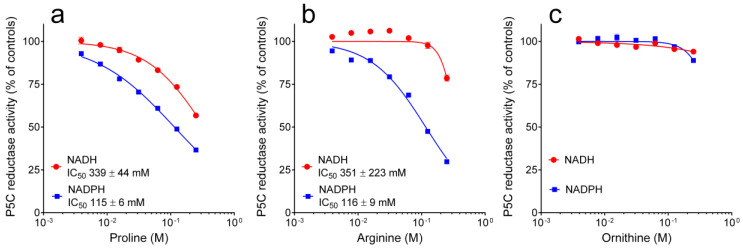
Effect of proline (**a**), arginine (**b**), and ornithine (**c**) on the activity of yeast P5C reductase. The purified enzyme was assayed in the absence or in the presence of increasing concentrations of a given amino acid, using either NADH or NADPH as the electron donor. Results were expressed as the percent of the activity obtained with untreated controls. For arginine and ornithine, added as hydrochlorides to prevent changes in the pH of the reaction mixture, controls received the same level of NaCl. Data are means ± SE over three replicates.

The possibility that enzyme products or related compounds may modulate enzyme activity was then considered. The addition of an increasing concentration of NADP^+^ up to 10 mM was found ineffective. On the contrary, NAD^+^ was slightly inhibitory in the 10^−4^ to 10^−2^ M range, with a significantly higher effect when NADPH was the electron donor (Figure 3). Similar patterns were found also with the other enzyme product, proline. In this case, 50% inhibition of enzyme activity was achieved at 115 mM proline when NADPH was the co-factor, whereas a 3-fold higher level was required to obtain the same result with NADH (Figure 4a). Interestingly, very similar data were evident when the reaction mixture was added with analogous levels of arginine. IC_50_ values were almost identical than with proline, while the differential effect was even more pronounced, in that arginine concentrations up to 100 mM were substantially ineffective when NADH was the co-factor (Figure 4b). The same concentrations of ornithine were not inhibitory, independently of the electron donor used (Figure 4c).

**Figure 5:**
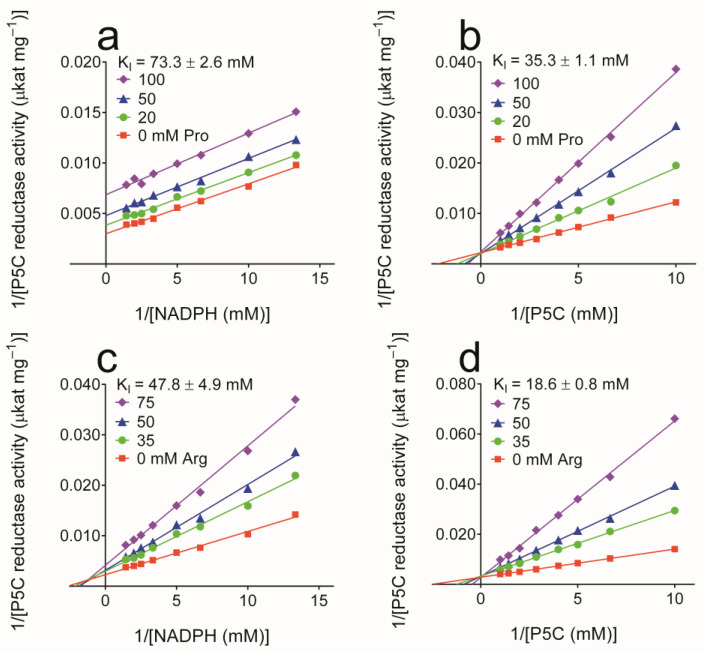
Kinetic analysis of the inhibition brought about by proline and arginine on the activity of yeast P5C reductase. Enzyme affinity was evaluated in the absence or in the presence of increasing concentrations of either amino acid. In the case of proline, reduced K_M_ and V_max_ (parallel lines in Lineweaver–Burk plot; panel **a**) and increased K_M_ with unaffected V_max_ (lines converging to the y-axis; panel **b**) accounted for an inhibition of uncompetitive and competitive type with respect to NADPH and P5C, respectively. The same pattern as with NADPH was obtained with NADH at higher proline doses. Concerning arginine, the same inhibition type was found with P5C (panel **d**). On the contrary, with NADPH K_M_ increased while V_max_ lowered (panel **c**), suggesting a mixed-type inhibition. The resulting K_I_ values are indicated, with that for proline against NADH equal to 534 ± 26 mM. The results are the mean over 3 technical replications. The whole experiment was repeated at least twice with different enzyme preparations, obtaining consistent results.

To obtain further information about the mechanism of enzyme inhibition, a kinetic analysis was performed. Results are presented in Figure 5. In the case of proline, reduced K_M_ and V_max_ and increased K_M_ with unaffected V_max_ accounted for an inhibition of uncompetitive and competitive type with respect to NADPH and P5C, respectively. The same pattern than with NADPH was obtained with NADH at higher proline doses (K_I_ = 534 ± 26 mM). Concerning arginine, the same competitive inhibition was found with respect to P5C. On the contrary, an increase in K_M_ and a decrease in V_max_ suggested an inhibition of mixed type with respect to NADPH.

**Figure 6:**
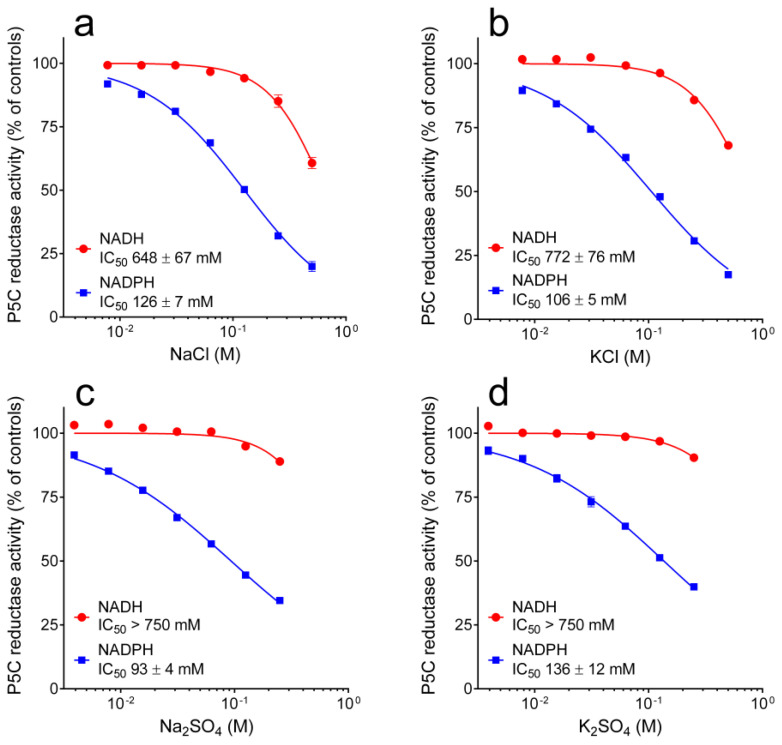
Effect of cations and anions on the activity of yeast P5C reductase. The purified enzyme was assayed in the absence or in the presence of increasing concentrations of chlorides (Panels **a** and **b**) or sulfates (Panels **c** and **d**), using either NADH or NADPH as the electron donor. Results were expressed as percent of the activity obtained with untreated controls. Data are means ± SE over three replicates.

Finally, the influence of increasing concentrations of salt was taken into account. Once again, a striking difference was found depending on which pyridine dinucleotide served as the electron donor. With NADH, the presence of salts up to 200 mM was completely ineffective. On the contrary, with NADPH as the co-factor, enzyme activity was inhibited by 50% at around 100 mM (Figure 6). The comparison of the patterns obtained with salts with the same anion and different cations (Figure 6a,b and Figure 6c,d), and vice versa (Figure 6a,c and Figure 6b,d), strongly suggested that the effect is primarily due to the anion.

**Figure 7:**
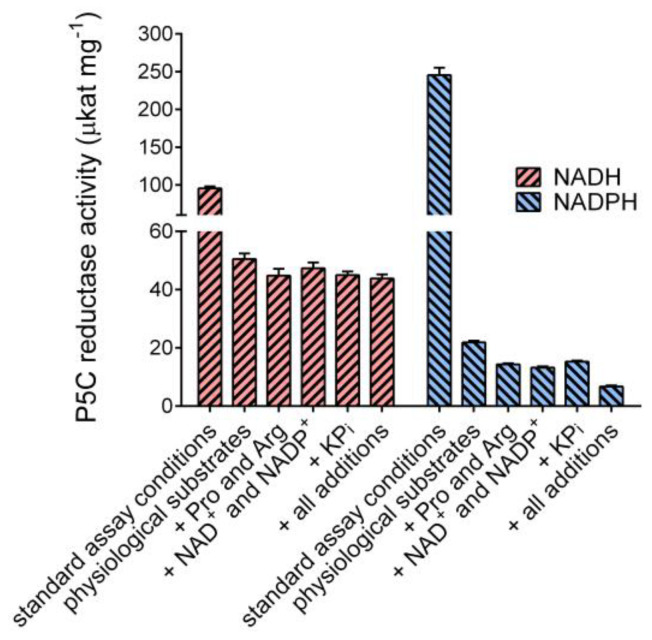
Influence of physiological concentrations of substrates and effectors on the activity of yeast P5C reductase. Enzyme activity was assayed under standard assay conditions (L-P5C 1 mM and NAD(P)H 0.5 mM), or in the presence of substrates and effectors at concentrations similar to those reported inside the yeast cell (L-P5C 100 μM, NADH 500 μM, NADPH 100 μM, NAD^+^ 1 mM, NADP^+^ 200 μM, Pro 1 mM, Arg 15 mM, and potassium phosphate 20 mM, pH 7.0), in a various array of combinations, as indicated. Results are mean ± SE over four replicates.

In order to understand whether such effects may have a significance in vivo, the activity of the purified enzyme was measured under standard assay conditions or in the presence of substrates, effectors, and ions at concentrations similar to those existing in the yeast cell [33]. The results are shown in Figure 7. Under near-saturating conditions, the activity with NADPH as the electron donor was 2.5-fold higher than that with NADH. On the contrary, at physiological levels of P5C (100 μM), NADH (500 μM), and NADPH (100 μM), the ratio was exactly reversed. Moreover, the presence of NAD^+^, NADP^+^, proline, arginine, and potassium phosphate did not significantly affect the NADH-dependent activity, whereas the NADPH-dependent rate was further reduced. When all effectors were present, as in the cell, the activity with NADH was 7-fold higher than that with NADPH.

### 3.3. Structural Features of Yeast P5C Reductase

**Figure 8:**
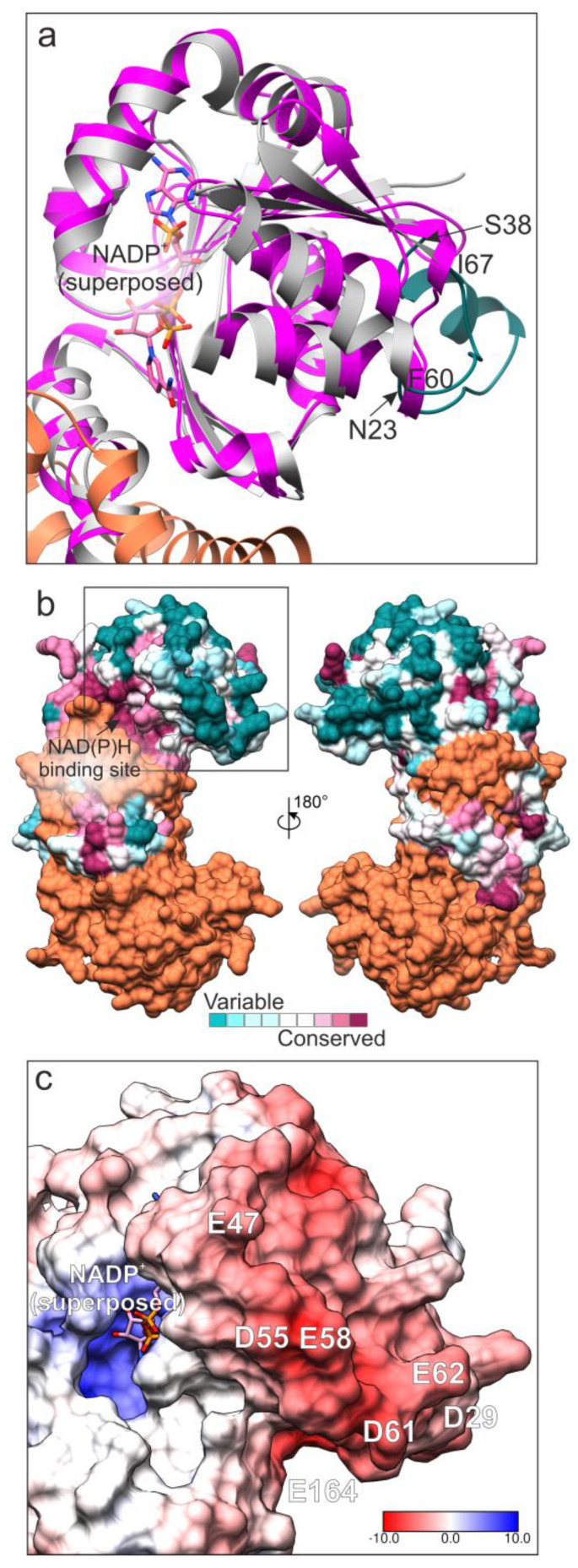
Analysis of the predicted structure of yeast P5C reductase. The three-dimensional model was built using the ColabFold implementation of AlphaFold [27]. A homodimer is shown for clarity, although the protein forms homodecamers. Panel **a** shows a comparative analysis of the ScPro3 model and the experimental structure of MtP5CR (magenta) in complex with NADP^+^ (PDB ID 5bsg, [35]). The variable fragments in the yeast enzyme (F60-I67 and N23-S38) are green. In (panel **b**), residue conservation is mapped on the ScP5CR surface for one subunit according to the color key (bottom); the second subunit is coral. The orientation on the left side in (panel **b**) is the same as in (panel **a**); the black rectangle indicates the fragment in (panels **a** and **c**). Surface electrostatic potential is shown in (panel **c**) for the same region as in (**a**); the color key is in kT/e. Negatively charged residues at variable positions are labeled.

In order to identify structural features that may account for the differences pointed out between the properties of yeast P5C reductase and the enzymes from other sources, an in silico analysis was carried out. A phylogenetic tree built with a multiple alignment for 728 P5C reductase amino acid sequences [24] and analyzed with the interactive Tree of Life (iTOL) software (version 6.4.3; [34]) allowed positioning the yeast enzyme among the predicted members of the P5C reductase family. It should be noted that the clade comprising all the sequences from fungi was found to be located at a considerable genetic distance from the clusters of other eukaryotes, and very close to the clade comprising most archea (Supplementary Figure S3). Given the lack of an experimental structure of *Sc*P5CR protein, we built its three-dimensional model using the ColabFold implementation of AlphaFold [27]. A homodimer was constructed to represent the complete active site and the swapped C-terminal fragment. Five best-scoring models of yeast P5C reductase were compared to each other to reveal the RMSD values between 0.19 and 0.28 Å. These low variations are consistent with the overall very high confidence of the structure prediction. Of note, the closest *Sc*P5CR homolog, whose structure is available in the Protein Data Bank (PDB), is the P5C reductase from the model plant *Medicago truncatula* (*Mt*P5CR) [35], with whom the protein shares 31% sequence identity (Supplementary Figure S4). Superposition of the *Sc*P5CR model with the experimental structure of *Mt*P5CR revealed the RMSD of 1.2 Å for 186 Cα pairs within a 2 Å distance. Most of the secondary structure elements overlap well, except for regions 23–38 and 60–67 in *Sc*P5CR that are substantially longer than the corresponding fragments in the *Mt*P5CR structure (Figure 8). Analysis of the residue conservation with Consurf [28] showed that the region 60–67 is particularly variable (Figure 8a,b).

When NADPH is the electron donor, arginine exhibited a mixed-type inhibition, suggesting that it may bind at an allosteric site, in addition to the active site. To propose the allosteric site, we analyzed the distribution of electrostatic potential on the *Sc*P5CR surface (Supplementary Figure S5). The analysis revealed negatively charged patches, which occur at surface regions of high sequence variability (Figure 8b,c). In particular, residues Asp29, Glu47, Asp55, Glu58, Asp61, Glu62, and Glu164, all of which are highly variable in other species, contribute to the negative charge on the *Sc*P5CR surface (Figure 8c). The molecular mechanism of the inhibition brought about by arginine cannot be inferred without the knowledge of its binding site. However, two conceivable scenarios include (i) inducing changes in the protein region that binds the 2′-phosphate of NADPH, or (ii) interfering with the protein flexibility by locking the hinge. Interestingly, ornithine was ineffective, suggesting that the site must specifically recognize the guanidine moiety. We also mapped the P5C binding site by superposing the structure of *Mt*P5CR in complex with proline (PDB ID 5bsh); proline is a mimic of P5C, likely binding at the same site. Proline/P5C most likely binds to the _248_GGTT_251_ motif in *Sc*P5CR that is highly conserved across species (Supplementary Figure S4). However, the putative allosteric binding site for arginine lies more than 25 Å away from that for proline/P5C. Therefore, independent binding of two arginine molecules at these two sites may contribute to the *Sc*P5CR regulation brought about by arginine.

**Figure 9:**
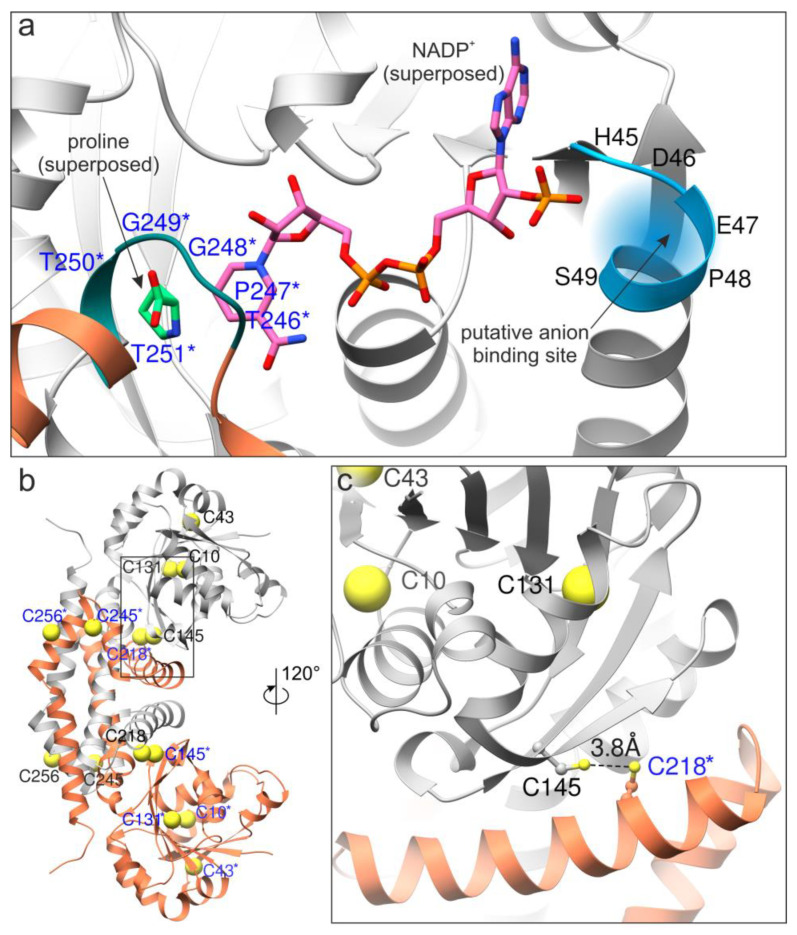
Identification of the putative sites of anion binding and disulfide formation. The coenzyme binding site with superposed NADP^+^ (from PDB ID 5bsg, [35]) and proline (PDB ID 5bsh) is shown in (panel **a**); the two subunits are gray and coral, respectively. Residues from the second subunit are labeled in blue and marked with asterisks. The anions most likely compete with the 2′-phosphate of NADPH. (Panel **b**) shows cysteine residues in *Sc*P5CR (Sγ atoms shown as yellow spheres), whereas a close-up over the Cys145-Cys218* pair (ball-and-stick) is presented in (panel **c**).

The superposition with the *Mt*P5CR structure in complex with NADP^+^ (PDB ID 5bsg) was also used to hypothesize on the possible anion binding site. Due to the different effectiveness depending on the pyridine dinucleotide used, the inhibiting anions most likely compete with the 2′-phosphate of NADPH by interacting with the _45_HDEPS_49_ fragment of *Sc*P5CR (Figure 9a). In this scenario, the helix dipole increases the positive charge on the protein surface.

The AlphaFold model of *Sc*P5CR also allowed the indication of cysteine pairs that could explain the stabilizing effect of DTT. The sequence of *Sc*P5CR contains seven Cys residues (positions 10, 43, 131, 145, 218, 245, and 256, Figure 9b). We then measured the Sγ-Sγ distances, allowing for inter-subunit pairing. In pairs Cys10-Cys43 and Cys245-Cys256, the Sγ atoms are further than 8 Å apart, strongly disfavoring a disulfide formation, unless a major conformational change takes place. However, in the Cys145-Cys218* (* indicates an element from the other subunit), the distance Sγ-Sγ was 3.8 Å. This suggests that a minor rearrangement, such as a change in the Cα-Cβ torsion angle, could enable an S-S bond formation (Figure 9c).

## 4. Discussion

The purification and a preliminary characterization of P5C reductase from bakers’ yeast were first reported in the 1980s [18,19]. It was described as a protein with a molecular weight of 125 kDa and a specific activity of about 47 μkat (mg protein)^−1^, showing a similar affinity toward NADH, NADPH, and P5C (K_M_ values of 48, 56, and 80 μM, respectively) but a V_max_ with NADPH that was about a half of that with NADH. The results we report are not in agreement with some of those conclusions. Retention patterns upon gel permeation chromatography suggested a remarkably larger native molecular mass (about 320 kDa) that would be consistent with a homodecameric composition of the holomer. Indeed, all crystallographic studies with full-length members of the P5C reductase superfamily showed to date only two oligomeric forms of the enzyme: dimeric (about 60 kDa) and decameric (about 300 kDa), whereas the occurrence of homotetramers has never been reported [24]. Concerning substrate affinity, a similar K_M_ value for P5C was found when NADH was the electron donor, but a much higher value was evident with NADPH. Only a single value had been reported in the cited early studies, most likely that with NADH as the co-factor. Conflicting results were obtained also with respect to the activity at saturating nicotinamide adenine dinucleotide concentrations, as, in our study, V_max_ with NADPH was not lower, but 2.5-fold higher than that with NADH. Although the presence of enzyme forms with dissimilar properties in different *S. cerevisiae* strains cannot be completely ruled out, a possible explanation for this discrepancy may be found in the sensitivity of the NADPH-dependent activity to salts. The presence of anions in the 10^−2^ to 10^−1^ M range was found to progressively reduce enzyme activity when NADPH was the electron donor, without affecting the NADH-dependent rate (Figure 6). Ion overloading into the reaction mixture may result from the contamination of the enzyme preparation and/or substrate solutions. In fact, in those early studies, the final protein fraction containing the enzyme, obtained from a DEAE-Sephadex column eluted with a gradient of 0–250 mM KCl, had not been desalted [18], and DL-P5C had been added following regeneration of its hydrazone in HCl [36] and subsequent neutralization, most likely by KOH addition.

In addition to correcting such errors, the present study provides information on other properties of the yeast enzyme, which have never been reported before and may contribute to a better understanding of its activity modulation in vivo. All members of the P5C reductase family have been reported to use both NADH and NADPH as the electron donor in vitro [24]. However, a thorough characterization of the enzyme from higher plants accounted for a likely preferential use of NADPH in vivo, although a significantly higher V_max_ was found at saturating concentrations of NADH [8,20,35]. Several features were in fact shown to limit the NADH-dependent activity under conditions similar to those expected in the cell. With NADH as the co-factor, enzyme activity was inhibited by NADP^+^ concentrations in the 10 to 100 μM range, by proline in the 10 to 100 mM range, and by anions at levels exceeding 100 mM, all conditions under which the NADPH-dependent activity was unaffected. On the contrary, cation concentrations higher than 10 mM were found to stimulate the latter up to 4-fold, contributing at least in part to stress-induced proline synthesis. Moreover, the apparent affinity for P5C was in all cases about 10-fold higher with NADH than with NADPH [8,20,35], with K_M_ values remarkably higher than the intracellular level of the substrate [37], which is suspected to exert toxic effects at millimolar concentrations [37,38]. Analogous results were reported for the enzyme from the human pathogen *Streptococcus pyogenes*, although, in that case, the sensitivity to salts was not investigated [22]. The properties found for yeast P5C reductase show an exactly opposite pattern. A significantly higher V_max_ is evident at saturating concentrations of NADPH, but the NADPH-dependent activity is inhibited by NAD^+^, proline, and anions, and shows an apparent K_M_ value for P5C (440 μM) largely exceeding the expected intracellular level of the substrate (50–100 μM). Under the same conditions, the NADH-dependent activity was substantially unaffected. On the whole, data, thus, seem to suggest a preferential use of NADH in vivo. Indeed, the activity with NADPH in the presence of substrates and effectors at concentrations similar to those reported in yeast cytoplasm [33] was only about 15% of that with NADH (Figure 7). Only for P5C reductase from the obligate fermentative bacterium *Zymomonas mobilis*, a preference for NADH has also been described to date, but in that case, it was a consequence of an intrinsically lower K_cat_/K_M_ ratio (2 × 10^5^ M^−1^ s^−1^ with NADH vs. 0.08 × 10^5^ M^−1^ s^−1^ with NADPH [23]). Contrary to the plant enzyme, the presence of salt did not stimulate the activity of *S. cerevisiae* P5C reductase, a result that seems consistent with the fact that yeast does not accumulate proline under hyperosmotic stress conditions [10].

Interestingly, the NADPH-dependent activity of yeast P5C reductase was found to be inhibited by millimolar concentrations of arginine, with an IC_50_ value almost identical to that of proline. To the best of our knowledge, this feature has not been reported to date for the enzyme from any other source, and is most likely a consequence of the dual role of P5C reductase in yeast, where it catalyzes the last reaction in both proline synthesis and arginine degradation [12]. A somehow similar mechanism has been reported for P5C synthetase, a bifunctional enzyme bearing both Pro2 and Pro1 activities, which is present in mammals in two forms deriving from the same primary transcript by differential splicing. The short form was found to be inhibited by ornithine (IC_50_ 0.25 mM), whereas the long version was unaffected, suggesting that the latter is responsible for the synthesis of proline, whereas the former is involved in the synthesis of ornithine and arginine via P5C and the reverse reaction of OAT [7]. In higher plants, which synthesize ornithine and arginine through a route that is distinct from that of proline [39], P5C synthetase was consistently found to be inhibited by ornithine and arginine only at concentrations significantly exceeding their homeostatic levels (IC_50_ > 50 mM [9]). In addition, yeast P5C reductase under substrate non-limiting conditions is inhibited by arginine at high doses (IC_50_ = 116 mM, Figure 4). However, the inhibition is of competitive type with respect to P5C, and a kinetic analysis at varying P5C concentration showed a K_I_ value of 19 mM (Figure 5), a concentration very near to that reported for arginine in the cell (15 mM [33]). These results strengthen the possibility that inhibition may take place in vivo, yet its significance could be questioned if considering that the rate of proline synthesis would not be reduced as a consequence, with the NADH-dependent activity being substantially unaffected. An opposite situation occurs in plants, where P5C reductase preferentially uses NADPH and is inhibited by proline only if NADH is the co-factor [8,20]. Although more experimental evidence is required to substantiate such a hypothesis, arginine inhibition may represent a mechanism to avoid NADPH depletion and maintain an optimal NADH/NADPH ratio under conditions in which high rates of P5C reductase activity are needed to use the amino acid as the main nitrogen or carbon source. NADPH levels in yeast are 5–10 fold lower than those of NADH [33], and recent data showed that perturbation of the subsequent mitochondrial oxidation of proline may in fact alter the redox balance [40]. In plants, on the contrary, when proline accumulation is needed to counteract an osmotic imbalance, inhibition of the NADH-dependent activity could be functional to avoid NADH depletion, with NADPH being continuously produced by the activation under stress of the oxidative pentose phosphate pathway.

The highest specific activity of yeast P5C reductase and its constitutive expression cause the presence of a limited amount of the enzyme in the cell. This, combined with its high lability during ammonium sulfate fractionation, restrained the yield of purified protein, and hampered the attempts to obtain crystals for X-ray diffraction. This notwithstanding, an in silico analysis allowed us to investigate the structural basis of at least part of enzyme features. Among the members of the P5C reductase family for which a crystallographic structure is available, the sequence of the yeast protein showed the highest similarity with that from the legume *M. truncatula*, despite exhibiting contrasting functional properties. The main structural difference is evident in the amino-terminal region (residues 23–38 and 60–67 in *Sc*P5CR; Figure 8, Supplementary Figure S4) that is substantially longer than that of the plant enzyme. In fact, these additional fragments contribute to form a negatively charged surface near the NADPH binding site that could represent the allosteric site for arginine interaction. Between these two additional sequences is also positioned the _45_HDEPS_49_ fragment, which may interact with anions that compete with the 2′-phosphate of NADPH (Figure 9a), thereby causing the specific inhibition of the NADPH-dependent activity. In silico analysis also allowed us to hypothesize on cysteine pairs that could explain enzyme lability and the stabilizing effect of reductants. The structure of yeast P5C reductase contains only two Cys residues that are near enough to consent disulfide formation, Cys145 and Cys218* (where * indicates an element from the other subunit forming a functional homodimer; Figure 9c). Such a linkage would lock the coenzyme-binding N-terminal domain in one conformation, suboptimal for catalysis. The addition of DTT to purified preparations prevented enzyme inactivation, but did not restore activity. Consistently, a secondary protein band with a retardation coefficient that is coherent with that of a homodimer was found upon SDS-PAGE analysis of purified preparations (Supplementary Figure S1).

## 5. Conclusions

The functional properties of yeast P5C reductase, only partially investigated in previous works, have been thoroughly determined. Contrary to the enzyme from plants, results account for a preferential use of NADH. Some unusual features, such as an allosteric inhibition by arginine and a lack of stimulation by salts, are in agreement with the peculiar role of proline metabolism in *Saccharomyces cerevisiae*, which does not accumulate proline under hyperosmotic stress and uses P5C reductase also for arginine catabolism.

## Figures and Tables

**Table 1:** Purification of *Saccharomyces cerevisiae* P5C reductase ^1^.

Step	Total Activity (nkat)	Protein (mg)	Specific Activity (nkat (mg protein)^−1^)	Purification (Fold)	Yield (%)
Crude extract	3234.8	85.6	37.79	1.0	100.0
60–80% ammonium sulfate fractionation	1169.1	16.71	69.96	1.9	36.1
Anion-exchange chromatography	787.4	0.0386	20,398	540	24.3
Gel permeation chromatography	505.3	0.0053	95,344	2523	15.6

^1^ Presented data are for a typical purification starting from 10 g of cell material. Activity was measured using NADH as the electron donor.

**Table 2:** Properties of *Saccharomyces cerevisiae* P5C reductase.

Specific activity _(NADH)_ ^1^	95.9 ± 2.7 μkat (mg protein)^−1^
Specific activity _(NADPH)_ ^1^	239.8 ± 6.8 μkat (mg protein)^−1^
pH optimum ^2^	6.61 to 7.34
K_M (app)_ for L-P5C _(NADH)_ ^3^	64.3 ± 5.5 μM
K_M (app)_ for L-P5C _(NADPH)_ ^3^	440.5 ± 21.4 μM
K_M (app)_ for NADH ^3^	84.0 ± 7.1 μM
K_M (app)_ for NADPH ^3^	133.4 ± 7.3 μM
V_max (NADH)_ ^3^	107.9 ± 3.0 μkat (mg protein)^−1^
V_max (P5C, with NADH as the co-substrate)_ ^3^	111.9 ± 2.2 μkat (mg protein)^−1^
V_max (NADPH)_ ^3^	294.2 ± 5.7 μkat (mg protein)^−1^
V_max (P5C, with NADPH as the co-substrate)_ ^3^	349.1 ± 8.1 μkat (mg protein)^−1^
K_cat (NADH)_ per monomer ^4^	3314 s^−1^
K_cat (NADPH)_ per monomer ^4^	10,515 s^−1^
K_cat_/K_M (NADH)_	3.95 × 10^7^ M^−1^ s^−1^
K_cat_/_KM (NADPH)_	7.88 × 10^7^ M^−1^ s^−1^

^1^ Specific activity was calculated under standard assay conditions (1 mM L-P5C and 0.5 mM NAD(P)H in 50 mM Tris-HCl buffer, pH 7.2) at 30 °C. Data are mean ± SE over three independent enzyme preparations. ^2^ The pH dependence of activity was evaluated by assaying the purified enzyme for up to 5 min in the presence of various amounts of Tris base to correct the pH following the addition of P5C in 1 M HCl. The actual pH value in the reaction mixture was measured at the end of the reaction with a microelectrode. pH optimum is defined as the pH interval in which the enzyme has >90% of maximal activity. ^3^ Apparent affinity constants and maximal catalytic rates were estimated from the plots obtained at varying NADH or NADPH concentration at a nearly saturating P5C level, and vice versa. Concentrations for the invariable substrate were 1 mM for L-P5C and 0.5 mM for NADH and NADPH. Variable substrates had a range of 60–600 μM and 100–1000 μM for the couple NADPH-P5C, and 40–400 μM and 50–700 μM for the couple NADH-P5C, respectively. Data and confidence intervals were computed using the corresponding functions in Prism 6 for Windows, version 6.03 (GraphPad Software, San Diego, CA, USA). ^4^ Catalytic constant was calculated from the computed maximal catalytic rate on the basis of a molecular weight of 30,132 Da, estimated from the gene sequence.

## Data Availability

The data that support the findings of this study are available from the corresponding author upon reasonable request.

## Supplementary Materials

The following supporting information can be downloaded at: https://www.mdpi.com/article/10.3390/microorganisms10102077/s1, Figure S1: Purification of yeast P5C reductase; Figure S2: Substrate affinity of yeast P5C reductase; Figure S3: Phylogenetic tree depicting the position of the yeast enzyme among the predicted members of the P5C reductase family; Figure S4: Multiple sequence alignment of *Saccharomyces cerevisiae* P5C reductase and selected representatives of the P5C reductase family; Figure S5: Distribution of electrostatic potential on the *Sc*P5CR surface.
