# Supplementary material for: Functional Characterization of Saccharomyces cerevisiae P5C Reductase, the Enzyme at the Converging Point of Proline and Arginine Metabolism
Source: Microorganisms. 2022 Oct 20;10(10):2077. doi: 10.3390/microorganisms10102077 (PMC9608600; doi:10.3390/microorganisms10102077)
Supplement: Supplementary file 1 [file microorganisms-10-02077-s001.zip › microorganisms-1965795-supplementary.pdf]

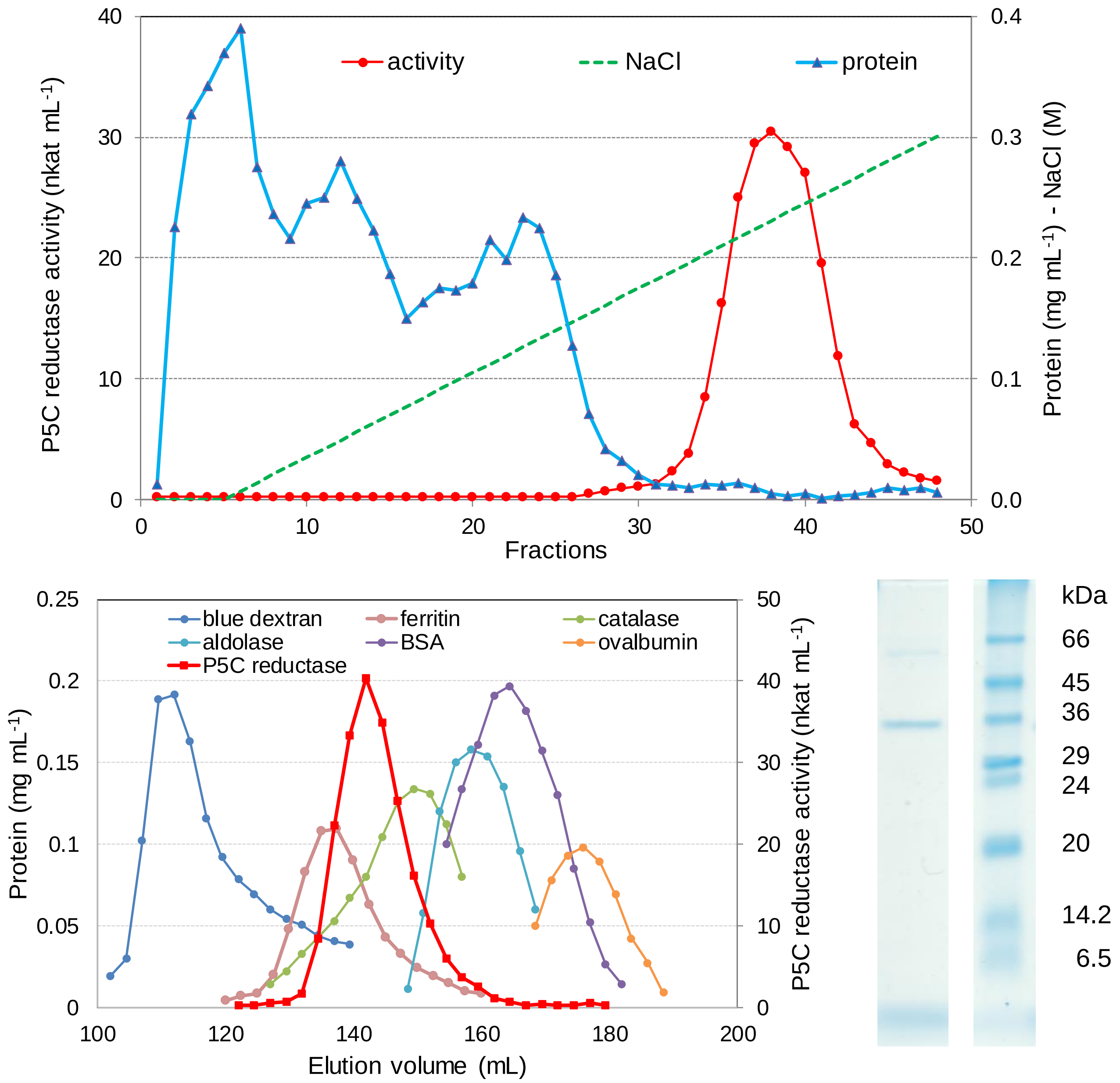

Supplementary Figure S1. Purification of yeast P5C reductase. Upper panel: anion exchange chromatography. Following ammonium sulfate fractionation of crude extracts, the 60-80% saturated fraction was desalted and loaded onto a DEAE-Sephacel column. Proteins were eluted with a linear gradient from 0 to 300 mM NaCl. Lower panel: gel permeation chromatography. Active fractions from the anion-exchange step were concentrated and loaded onto a Sephacryl S300 column. Fractionation on the same column of suitable molecular markers, as indicated, allowed an estimate of the native molecular mass of the enzyme. Active fractions from the gel permeation step were concentrated and analysed by SDS-PAGE. A protein with the expected mobility, as judged by comparison with the retardation coefficient of molecular markers (SigmaMarkers M3913) run in a parallel lane, was evident. A second, fainter band was also present that, based on the estimated relative mass (about 60 kDa), most likely represents a homodimer of the main protein.

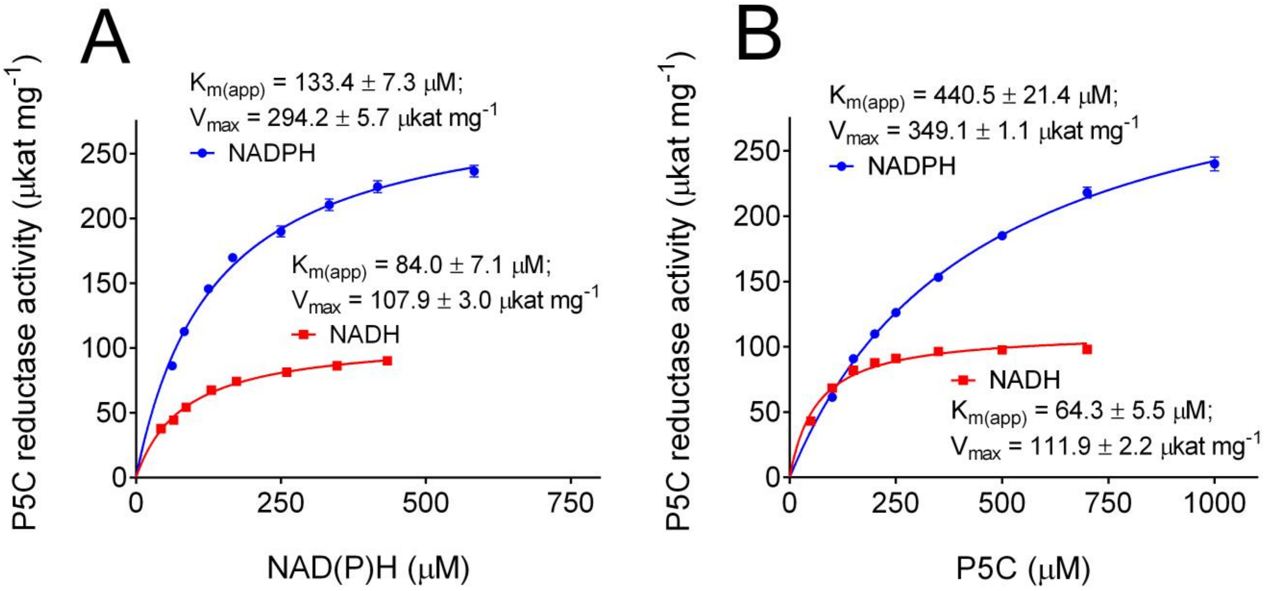

Supplementary Figure S2. Substrate affinity of yeast P5C reductase. Michaelis-Menten graphs were obtained at varying NAD(P)H (Panel A) or L-P5C (Panel B) concentration while maintaining constant the other substrate (1 mM L-P5C or 0.5 mM NAD(P)H, respectively). Non-linear regression of data allowed calculation of K_M and V_{max} values, as indicated. Data are mean ± SE over three replicates. The experiment was repeated twice with independent enzyme preparations, and consistent results were found.

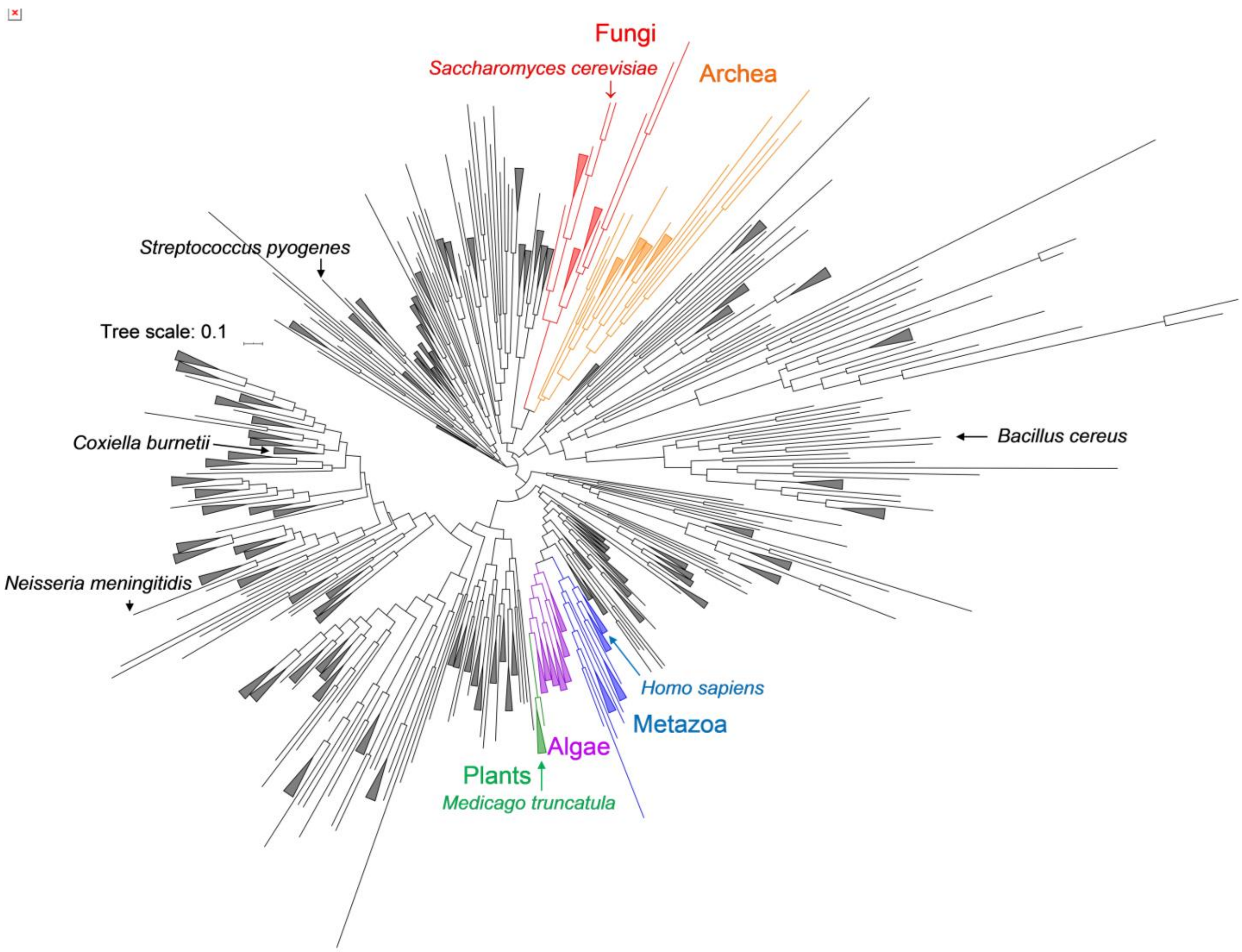

Supplementary Figure S3. Phylogenetic tree depicting the position of the yeast enzyme among the predicted members of the P5C reductase family. The maximum-likelihood phylogenetic unrooted tree built with a multiple alignment for 728 P5C reductase amino acid sequences [1] was analysed with the interactive Tree of Life (iTOL) software (version 6.4.3; [2]) with the following options: circular mode, auto collapse clade 0.7 > avg. BLR. The collapsed node with 3 leaves comprising the *S. cerevisiae* enzyme was expanded. Color code: eubacteria: black; archea: orange; fungi: red; eukaryotic algae: purple; plants: green; metazoa: blue. The organism name and the position of P5C reductase proteins with solved crystallographic structure are also indicated.

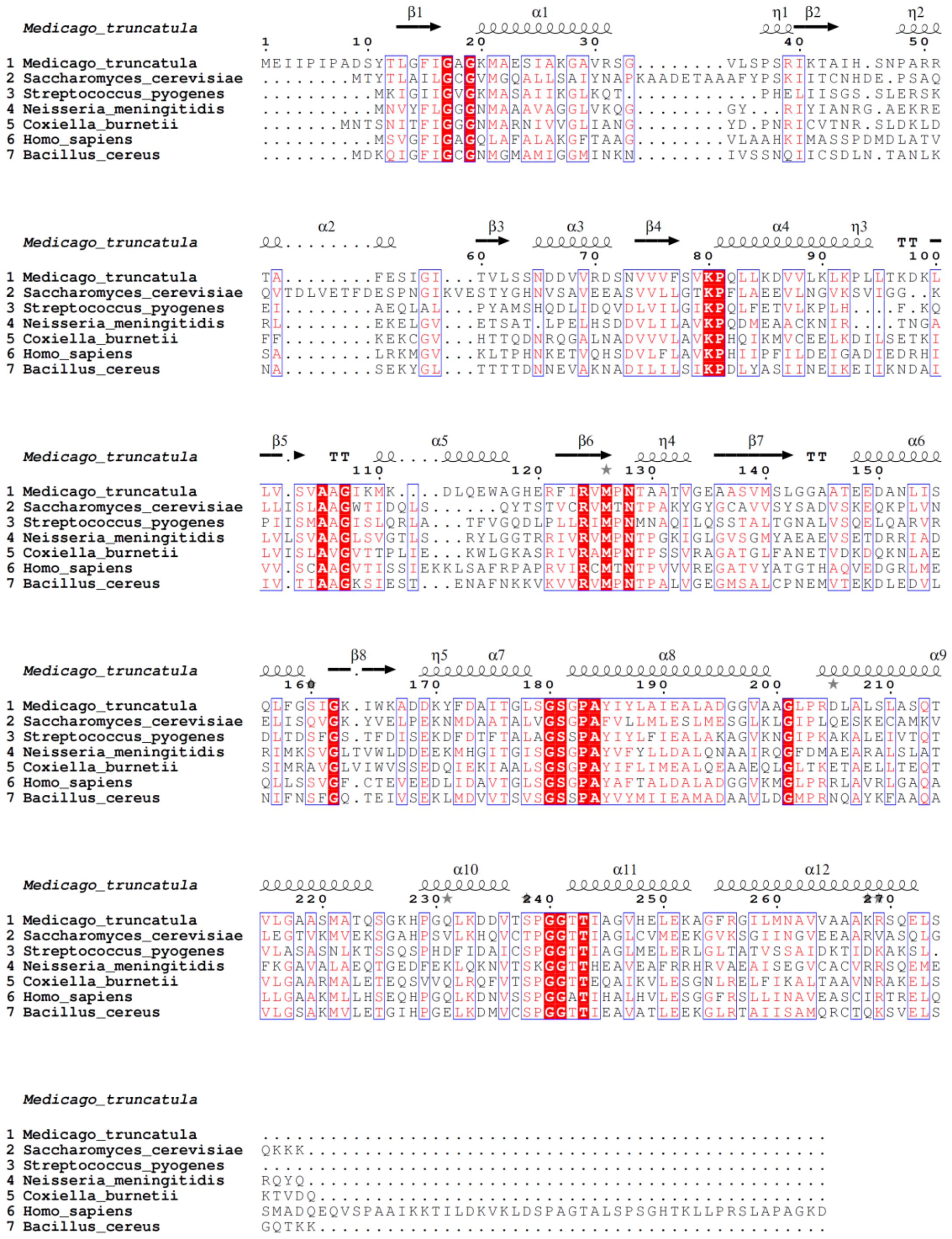

Supplementary Figure S4. Multiple sequence alignment of *Saccharomyces cerevisiae* P5C reductase and selected representatives of the P5C reductase family. The proteins have been aligned using Clustal Omega (https://www.ebi.ac.uk/Tools/msa/clustalo/) and Espript 3.0 (https://espript.ibcp.fr/ESPript/ESPript/) Sequence identities are emphasized in red and similarities are highlighted as red letters. The corresponding secondary structure of *Medicago truncatula* P5C reductase, the P5C reductase showing the highest sequence similarity with the yeast protein (among P5C reductases whose experimental 3D structures have been determined), is shown on the top. Helices appear as scribble, β-strands as arrows. The PDB ID numbers of the sequences used are: *Homo sapiens* 2IZZ; *Neisseria meningitides* 1YQG; *Streptococcus pyogenes* 2AHR; *M. truncatula* 5BSE; *Coxiella burnetii* 3TRI; *Bacillus cereus* 3GT0.

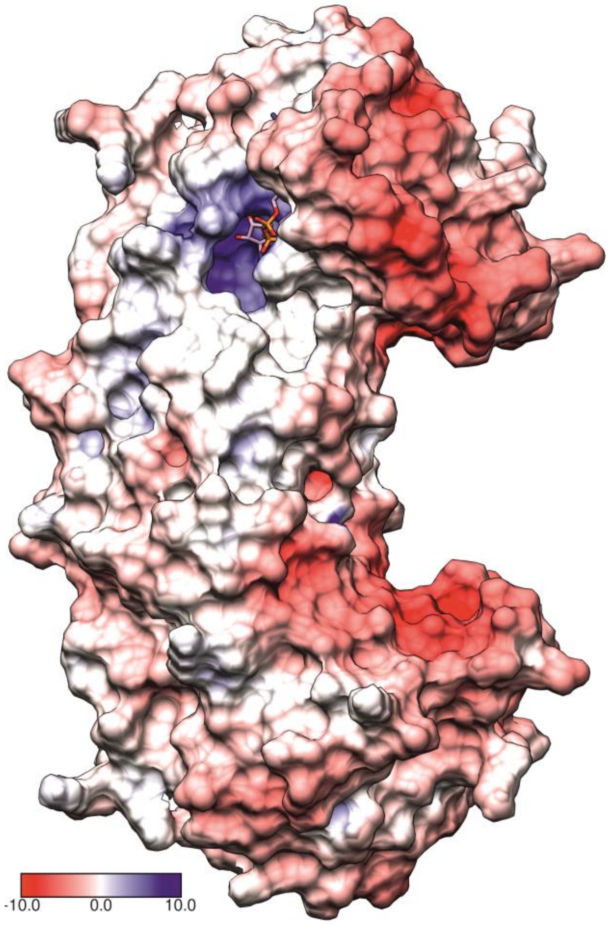

Supplementary Figure S5. Distribution of electrostatic potential on the *Sc*P5CR surface. Only the dimer is presented for clarity. The color key (bottom-left) is in kT/e. The partially visible $NADP^+$ is superposed from *M. truncatula* P5CR (PDB ID 5bsg, [3]).

References

1. Forlani, G.; Makarova, K.S.; Ruszkowski, M.; Bertazzini, M.; Nocek, B. Evolution of plant δ^1-pyrroline-5-carboxylate reductases from phylogenetic and structural perspectives. *Front. Plant Sci.* **2015**, *6*, 567, doi:10.3389/fpls.2015.00567.
2. Letunic, I.; Bork, P. Interactive Tree Of Life (iTOL) v5: an online tool for phylogenetic tree display and annotation. *Nucleic Acids Res.* **2021**, *49*, W293-w296, doi:10.1093/nar/gkab301.
3. Ruszkowski, M.; Nocek, B.; Forlani, G.; Dauter, Z. The structure of *Medicago truncatula* δ^1-pyrroline-5-carboxylate reductase provides new insights into regulation of proline biosynthesis in plants. *Front. Plant Sci.* **2015**, *6*, 869, doi:10.3389/fpls.2015.00869.
